# Association between use of antihypertensive drugs and the risk of cancer: a population-based cohort study in Shanghai

**DOI:** 10.1186/s12885-023-10849-8

**Published:** 2023-05-11

**Authors:** Suna Wang, Li Xie, Jianlin Zhuang, Ying Qian, Guanglu Zhang, Xiaowei Quan, Lei Li, Herbert Yu, Weituo Zhang, Wensui Zhao, Biyun Qian

**Affiliations:** 1 School of Public Health and Hongqiao International Institute of Medicine, Shanghai Tongren Hospital, Shanghai Jiao Tong University School of Medicine, NO.720 Xianxia Road, Changning District, Shanghai, 200050 China; 2 Clinical Research Institute, Shanghai Jiao Tong University School of Medicine, Shanghai, 200025 China; 3 Shanghai Changning District Center for Disease Control and Prevention, NO. 39, Yunwushan Road, Changning District, Shanghai, 2000040 China; 4 Shanghai Clinical Research Promotion and Development Center, Shanghai Hospital Development Center, Shanghai, 200041 China; 5 Clinical Research Center, Shanghai Children’s Medical Center, Shanghai Jiao Tong University School of Medicine, Shanghai, 200127 China; 6 Cancer Epidemiology Program, University of Hawaii Cancer Center, 701 Ilalo Street, Honolulu, HI 96813 USA

**Keywords:** Antihypertensive drugs, Cohort study, Cancer risk, Incidence, Electronic health records

## Abstract

**Background:**

Previously studies shown a potential risk of antihypertensive medicines in relation to cancer susceptibility, which creating significant debate in the scientific community and public concern. We sought to investigate the relationship between antihypertensive medicines and cancer risk, by drug type and class.

**Methods:**

We conducted a population-based cohort study and enrolled patients diagnosed with hypertension from community healthcare centers in Changning District, Shanghai, China. Antihypertensive drug administration were classified as five common antihypertensive drugs. The main outcomes were incidence of total cancer and by major cancer type.

**Results:**

Between January 2013 and December 2017, a total of 101,370 hypertensive patients were enrolled in this cohort. During a mean follow-up of 5.1 (SD 1.3) years, 4970 cancer cases were newly diagnosed in the cohort. CCBs were the most frequently used antihypertensives which were associated with a moderately increased risk of total cancer (hazard ratio, HR = 1.11, 95% CI: 1.05–1.18). The second commonly used drug ARBs were also associated with increased risk of total cancer (HR = 1.10, 95%CI: 1.03–1.17) as well as lung and thyroid cancers (HR = 1.21, 95%CI: 1.05–1.39; HR = 1.62 95%CI: 1.18–2.21, respectively). No significant association was found between cancer and other antihypertensives. Hypertensive patients who use more than one class of antihypertensives drugs had a higher risk of total cancer (HR: 1.22, 95%CI: 1.10–1.35 for two classes; HR: 1.22, 95%CI: 1.03–1.45 for three or more classes), and a possible dose–response relationship was suggested (*P* for trend < 0.001). The risk of thyroid cancer was higher in hypertensive patients prescribed with three or more antihypertensive classes.

**Conclusions:**

Use of ARBs or CCBs may be associated with an increased risk of total cancer. Taking more than one class of antihypertensives drugs appeared to have a higher risk for total cancer.

**Supplementary Information:**

The online version contains supplementary material available at 10.1186/s12885-023-10849-8.

## Introduction

Arterial hypertension is one of the major causes of mortality and morbidity in the world. The condition requires long-term use of antihypertensives which can last for decades, and this long-term administration makes antihypertensives one of the most-frequently prescribed drugs [1]. Therefore, it is critical to carefully monitor and detect adverse effects of the drugs because even minor effects may have significant ramifications on a wide scale.

Experimental studies indicate that angiotensin-converting enzyme inhibitors can promote bradykinin and substance P and mediates tumor growth. Angiotensin II receptors have been found to have a role in regulating tumor proliferation, migration, and angiogenesis. If the suspected effects of antihypertensive drugs on tumorigenesis cannot be ruled out,, misunderstanding will have a negative impact on the success of hypertension management [2].

A recent meta-analysis of 33 randomized controlled trials have found no evidence of antihypertensive use in promoting cancer, but the limited follow-up with median less than 5 years and non-targeted design for cancer restricted the extension and the risk of site-specific cancer was not displayed [3, 4]. A number of epidemiological studies focused on the association between antihypertensive treatment and risk of cancer including overall and site-specific risk, and no consistent conclusions could be drawn from these studies [5–7]. While some studies revealed increased risks of total or site-specific cancers in association with the use of antihypertensives, others reported null associations or reduced risk [8, 9]. These conflicting results may attribute to varied durations of follow-up, different races and sample sizes, distinctive comparators, and diverse study designs. Moreover, few studies have investigated the combined effects of using multiple classes of antihypertensive drugs as many patients are treated under polytherapy [10].

In this study, we aimed to explore the ongoing controversy about the safety of blood pressure-lowering medication with respect to cancer risk, as well as assessing the cumulative risk of ever use of any classes of antihypertensive drugs.

## Methods and materials

### Study population

**Fig. 1:**
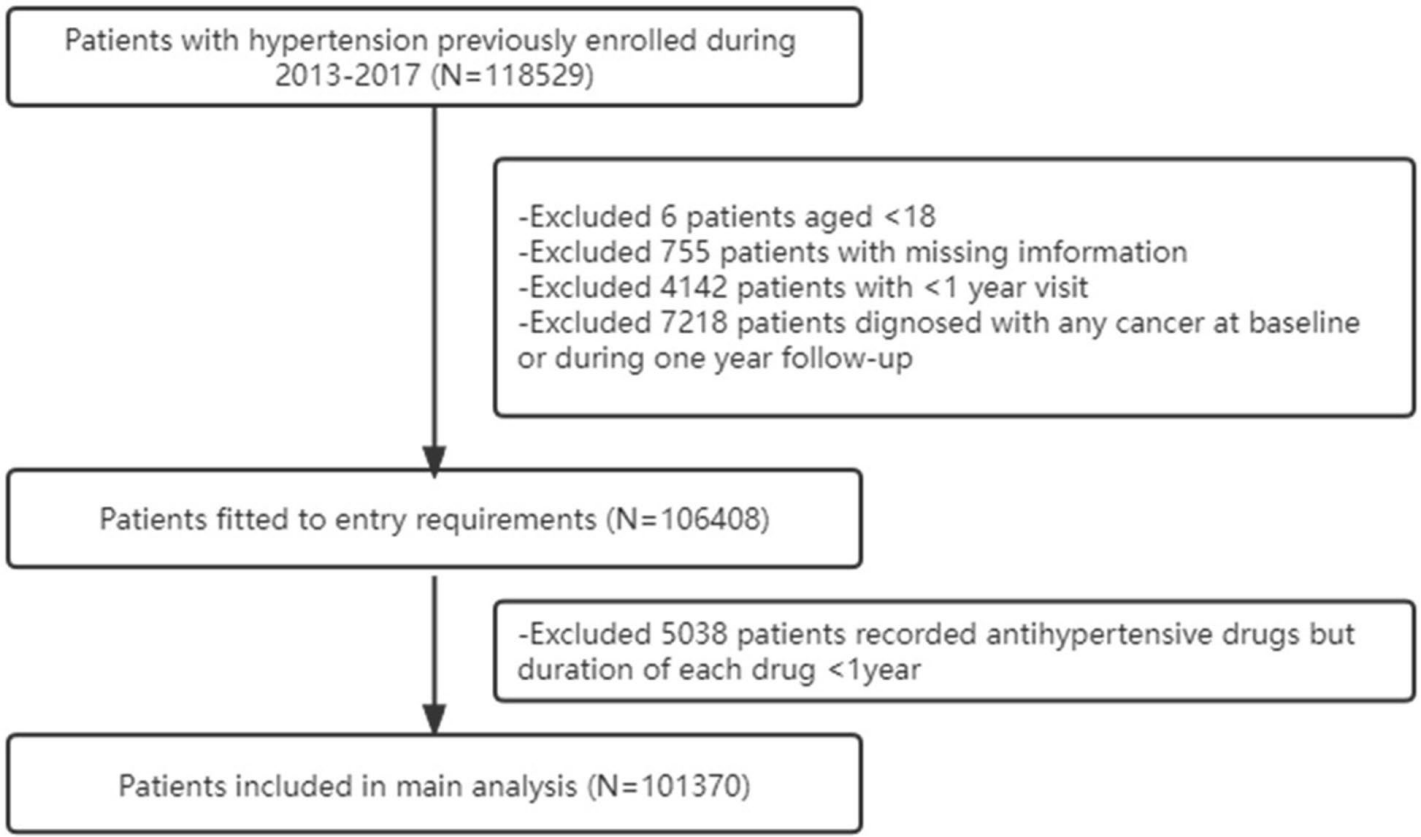
Flow chart of enrollment of study participant

The present study was a longitudinal, community‐based study of hypertensive patients, carried out within the framework of Shanghai Primary Public Health Service Program by Center of Diseases Prevention and Control of Shanghai. From 2013 to 2017, patients who previously diagnosed with hypertension by doctors were invited to participate in the Shanghai Primary Health Care Hypertension Management by community healthcare centers in Shanghai Changning. All participants were permanent residents of Shanghai Changning District aged ≥ 18 years, followed every 3 months since enrollment. In each visit, demographic information and hypertension related data including drug medication as well as blood pressure were collected by professional family doctors. This study was approved by the ethic review committee at Shanghai Tongren Hospital (No.2021–019-01) and conducted in accordance with Declaration of Helsinki. The ethics review committee approved the waiver of informed consent from participants due to the nature of the study involving electronic health record systems. Details of flow chart are shown in Fig. 1.

### Exposure assessment

**Fig. 2:**
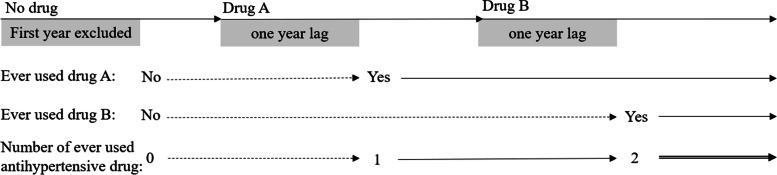
Assignation of time-dependent variable by ever-exposed to drug and number of ever used drug classes during follow-up

At each visit, hypertensive patients were asked about their antihypertensive medication use by community physicians. The major antihypertensive drugs were classified as angiotensin-converting enzyme inhibitors (ACEIs), angiotensin receptor blockers (ARBs), beta blockers (BBs), calcium channel blockers (CCBs), and diuretics [11, 12]. Individuals who described the same drug class for at least two consecutive records and duration of drug use for more than one year according to the first and last prescription times were enrolled as exposure to this drug class and considered drug users. Use of antihypertensive drugs for less than a year was considered a short-term use, and the impact of short-term use of antihypertensive drugs is likely to be small or limited. Patients who did not report to take any antihypertensive drugs for at least a year during the study period were considered no exposure or non-users. If participants who were prescribed antihypertensive drugs, but the duration of each class was less than one year, were not considered to expose or not expose to any drug class and therefore were excluded from the study. The exposure of each class of antihypertensive drugs was regarded as a time-dependent variable which meant that the participants would be in an unexposed condition before the first record of drug exposure. In order to reduce the possibility of reverse causality, we considered the start of exposure one year after the first date recorded for drug use. When examining the number of classes of antihypertensive drugs ever used, we also treated the class number as a time-dependent variable which was classified into 0, 1, 2, ≥ 3 based on the above rules. Details of exposure definition are shown in Fig. 2.

### Outcome assessment

We performed a data linkage to Shanghai Cancer Registry to identify newly diagnosed cancer cases in our cohort. Follow-up time began at one year after enrollment and ended at the first event of a diagnosis of any cancer, death or the terminate of this study (Last follow-up date: December 31, 2019). Also, the newly diagnosed cancer patients were identified through data linkage to Shanghai Cancer Registry (Data cut-off date: December 31, 2019). We analyzed the four most common cancer sites in Shanghai [13]. According to the 10th International Classification of Diseases (ICD10), we identified lung cancer (C34), colorectal cancer (C18-20), thyroid cancer (C73), and stomach cancer (C16).

### Potential confounders

All covariates were measured at baseline when the participants were enrolled in the cohort. The following confounders were adjusted in multi-variable analysis: age, sex, body mass index (BMI; < 18.5, 18.5–24, ≥ 24), cigarette smoking (non-smoker, ever smoker, current smoker), alcohol drinking (non-drinker, ever drinker, or current drinker), physical activity (never, sometimes, always, everyday), diabetes, and coronary artery heart disease (CHD). Chronic obstructive pulmonary disease (COPD) was added as a confounding factor in analysis of lung cancer. Blood pressure was measured at each visit on the right arm with an electronic sphygmomanometer after participants rested quietly for at least 5 min. The final multivariate models were also adjusted for the mean of systolic blood pressure (SBP), which was defined as a categorical variable using the cut-off of 130 mmHg according to 2020 International Society of Hypertension (SBP < 130 mmHg, SBP ≥ 130 mmHg). Missing value of above potential confounders was less than 2% (BMI, *N* = 165; smoking, *N* = 21; alcohol, *N* = 21; physical activity, *N* = 10), so that we excluded patients without confounder data at baseline.

### Statistical methods

To examine the associations between cancer risk and use of specific antihypertensive drugs, we applied time-dependent Cox proportional hazards regression models by calculating hazard ratios (HR) with their 95% confidence intervals (CI), after checking the assumption of proportional hazards by the Schoenfeld test. For analysis of a specific cancer site, we censored the follow-up times at the first diagnosis of any other cancers. In analysis of specific drug classes, we focused only on hypertensive patients who administered the drug class of interest and compared them to those who did not administer that antihypertensive drug. We further repeated analysis stratified by the mean of SBP to assess potential effect modification by blood pressure. Several sensitivity analyses were conducted to evaluate the robustness of findings. Firstly, we extended the length of latency period by excluding cancer cases who were diagnosed within two years of enrollment. Secondly, given uncertainties on the minimum length of exposure, we increased the exposure time of drug use for at least two years. Finally, we repeated the analysis by including the mean of SBP as a continuous covariate to control the potential influence of blood pressure. All data analyses were performed using R-4.0.3 and package “survival” was used for Cox regression models. *P* value < 0.05 was defined as statistical significance. Considering the effect of multiple testing, we set a significance level of *P* < 0.01 as a Bonferroni adjustment for the five classes of drugs tested.

## Results

### Characteristics of study population

We enrolled 118528 hypertensive patients during initially screened, then we excluded participants with less than one-year visit or being diagnosed with any cancer at baseline. Also, we further excluded those suffering from cancer within one year of follow-up to minimize potential reverse causality between cancer risk and drug use (Fig. 1).

Among 101,370 participants in this study, with a mean follow-up of 5.1 (SD 1.3) years beyond the first-year exclusion, 4,970 individuals were newly diagnosed with cancer during the follow-up time. The mean age of the participants at enrollment was 68 years, and 46% of them were male. As showed in Table 1, the most popular antihypertensive drugs prescribed with a duration exceeding one year were CCBs (41,598, 41%) and ARBs (38,580, 38%), and people who used both were about 40%, followed by diuretics (8,107, 8%), BBs (7,837, 7%), and ACEIs (5,981, 6%). There were 51,487 (50%) participants who only used one antihypertensive drug and 22,771 (22%) who used two or more antihypertensive classes. The details of antihypertensive drug use showed in Supplement Figure 1. ACEIs users were more likely to be single users, and diuretics users were likely to be multiple users. Participants who ever used common antihypertensive drugs tended to be older, to be male and to have higher body mass index, with BBs users showing the highest age, ACEIs users showing the most prevalence of male, and diuretics users showing the highest BMI. More current smokers and current drinkers were seen to use antihypertensives, while they exercised more frequently. Diabetes and CHD were more common among those who used antihypertensives particularly in BBs users, but COPD showed no difference. Although there were only about 34% individuals who displayed higher blood pressure among the entire cohort, indicating well controlled blood pressure in the population, participants who ever used common antihypertensive drugs had slightly higher SBP while most diuretics users displayed elevated SBP.

**Table 1:** Characteristics of participants by treatment use of five common antihypertensive drugs

Characteristic	Never used	Ever used					
		**Any**	**ACEIs**	**ARBs**	**BBs**	**CCBs**	**Diuretics**
*N*	27112	74258	5981	38580	7837	41598	8107
* Age (mean (SD))*	66.96 (12.35)	68.59 (11.21)	68.91 (11.21)	68.23 (11.19)	69.68 (10.75)	68.99 (11.01)	69.09 (11.26)
* Sex: male (%)*	12245 (45.2)	34148 (46.0)	3156 (52.8)	17393 (45.1)	3648 (46.5)	19271 (46.3)	3654 (45.1)
* SBP mean:* ≥ *130(%)*	8982 (33.1)	25686 (34.6)	2070 (34.6)	13763 (35.7)	2648 (33.8)	14674 (35.3)	3085 (38.1)
*BMI (%)*							
< *18.5*	676 (2.5)	1443 (1.9)	144 (2.4)	673 (1.7)	183 (2.3)	790 (1.9)	141 (1.7)
* 18.5–24*	14349 (52.9)	34072 (45.9)	2770 (46.3)	17328 (44.9)	3512 (44.8)	18907 (45.5)	3442 (42.5)
≥ *24*	12087 (44.6)	38743 (52.2)	3067 (51.3)	20579 (53.3)	4142 (52.9)	21901 (52.6)	4524 (55.8)
*Smoking (%)*							
* Never*	23496 (86.7)	61688 (83.1)	4862 (81.3)	32233 (83.5)	6547 (83.5)	34160 (82.1)	6647 (82.0)
* Ever*	666 (2.5)	2618 (3.5)	238 (4.0)	1382 (3.6)	368 (4.7)	1539 (3.7)	313 (3.9)
* Current*	2950 (10.9)	9952 (13.4)	881 (14.7)	4965 (12.9)	922 (11.8)	5899 (14.2)	1147 (14.1)
*Alcohol (%)*							
* Never*	23207 (85.6)	62730 (84.5)	5034 (84.2)	32695 (84.7)	6704 (85.5)	34944 (84.0)	6814 (84.1)
* Ever*	2556 (9.4)	7069 (9.5)	567 (9.5)	3698 (9.6)	715 (9.1)	3986 (9.6)	765 (9.4)
* Current*	1349 (5.0)	4459 (6.0)	380 (6.4)	2187 (5.7)	418 (5.3)	2668 (6.4)	528 (6.5)
*Sports (%)*							
* Never*	10051 (37.1)	26582 (35.8)	2106 (35.2)	14042 (36.4)	2657 (33.9)	14551 (35.0)	2934 (36.2)
* Sometimes*	6401 (23.6)	18428 (24.8)	1538 (25.7)	9503 (24.6)	1957 (25.0)	10354 (24.9)	1964 (24.2)
* Always*	7084 (26.1)	21334 (28.7)	1840 (30.8)	10718 (27.8)	2418 (30.9)	12317 (29.6)	2412 (29.8)
* Everyday*	3576 (13.2)	7914 (10.7)	497 (8.3)	4317 (11.2)	805 (10.3)	4376 (10.5)	797 (9.8)
* No. of diabetes (%)*	3335 (12.3)	13604 (18.3)	1208 (20.2)	7528 (19.5)	1630 (20.8)	8148 (19.6)	1606 (19.8)
* No. of CHD (%)*	3147 (11.6)	12908 (17.4)	1136 (19.0)	6837 (17.7)	2095 (26.7)	7275 (17.5)	1421 (17.5)
* No. of COPD (%)*	239 (0.9)	655 (0.9)	48 (0.8)	342 (0.9)	83 (1.1)	364 (0.9)	56 (0.7)
*No. of ever used antihypertensives (%)*							
* 1*	-	51487 (69.3)	3289 (55.0)	20071 (52.0)	2197 (28.0)	24811 (59.6)	1119 (13.8)
* 2*	-	18192 (24.5)	2030 (33.9)	14154 (36.7)	3566 (45.5)	12631 (30.4)	4003 (49.4)
≥ *3*	-	4579 (6.2)	662 (11.1)	4355 (11.3)	2074 (26.5)	4158 (10.0)	2985 (36.8)

*ACEIs* Angiotensin-converting enzyme inhibitors, *ARBs* Angiotensin receptor blockers, *BBs* Beta blockers, *CCBs* Calcium channel blockers, *SBP* Systolic blood pressure, *BMI* Body mass index, *CHD* Coronary artery heart disease, *COPD* Chronic obstructive pulmonary disease

### Effect of using single antihypertensive drug

**Fig. 3:**
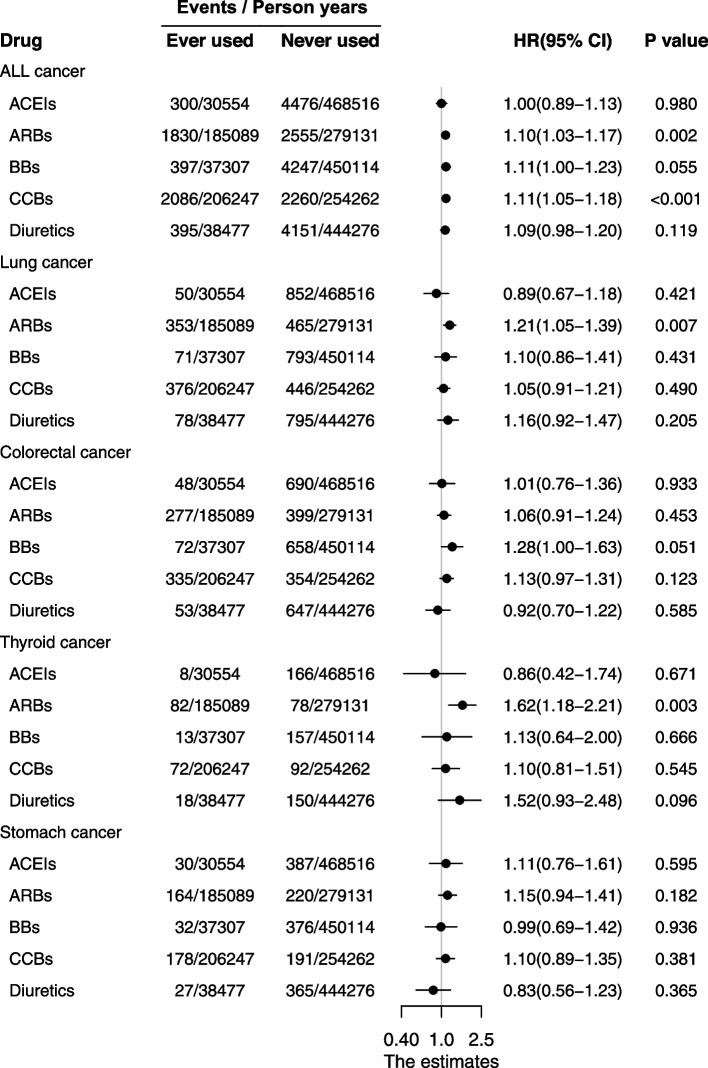
Hazards ratio for total and specific cancer associated with ever use of antihypertensive drugs. *HR* hazard ratio. *Adjusted for age, sex, body mass index, cigarette smoking, alcohol drinking, physical activity, diabetes, coronary artery heart disease and SBP mean. Chronic obstructive pulmonary disease included in lung cancer

Compared to non-users, ARBs users and CCBs users were associated with 10% (95%CI: 1.03–1.17, *P* = 0.002) and 11% (95%CI: 1.05–1.18, *P* < 0.001) higher risks of total cancer, respectively (Fig. 3). No association was found between cancer risk and use of ACEIs, BBs, or diuretics. For specific cancer sites, we observed significant increase in risk of lung cancer and thyroid cancer in association with the use of ARBs, hazard ratios of 1.21 (95% CI: 1.05–1.39, *P* = 0.007) and 1.62 (95% CI: 1.18–2.21, *P* = 0.003) after multiple testing, respectively. No association was showed with any other cancer sites in people using other antihypertensive drugs. As for stratified analysis, CCBs users were significantly positive associated with the risk of total cancer (HR: 1.10, 95%CI: 1.02–1.19, *P* = 0.009), while ARBs users showed a borderline statistically significant increased risk of total cancer (HR: 1.10, 95%CI: 1.02–1.18, *P* = 0.012) and an evident statistically significant increased risk of thyroid cancer (HR: 1.79, 95%CI: 1.26–2.54, *P* = 0.001) in hypertension with SBP < 130 mmHg (Supplement Figure 2). In hypertension with SBP ≥ 130 mmHg, there was no significant association between antihypertensive drug and cancer risk after multiple testing, although CCBs users showed a borderline statistically significant increased risk of total cancer (HR: 1.14, 95%CI: 1.03–1.26, *P* = 0.015).

### Cumulative effect of using multiple classes of antihypertensive drugs

**Fig. 4:**
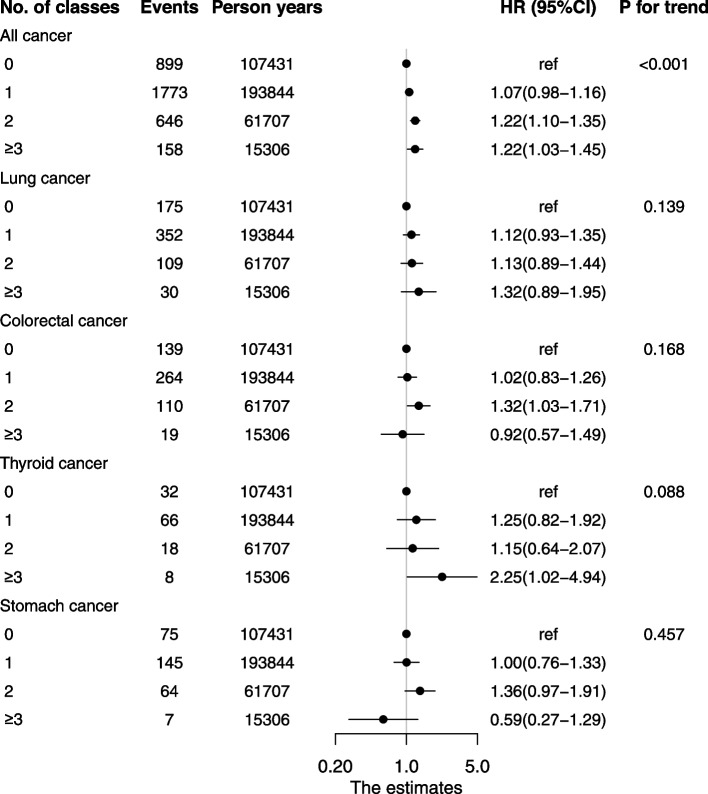
Association between cumulative number of ever-used antihypertensive drugs and types of cancer. *HR* hazard ratio. *Adjusted for age, sex, body mass index, cigarette smoking, alcohol drinking, physical activity, diabetes, coronary artery heart disease and SBP mean. Chronic obstructive pulmonary disease included in lung cancer

Hypertensive patients who used two or more classes of antihypertensive drugs had higher risk of total cancer compared to those who never used any antihypertensive drugs (HR: 1.22, 95%CI: 1.10–1.35 for two classes; HR: 1.22, 95%CI: 1.03–1.45 for three or more classes, Fig. 4), and the trend was statistically significant (*P* < 0.001). Significant associations were not found for 4 common cancer sites (*P* = 0.139 for lung cancer; *P* = 0.168 for colorectal cancer; *P* = 0.088 for thyroid cancer; and *P* = 0.457 for stomach cancer), except for thyroid cancer which showed increased risk associated with use of three or more classes of drugs (HR: 2.25, 95%CI: 1.02–4.94).

### Sensitivity analyses

When we excluded cancer cases diagnosed within 2 years of enrollment from our initial analysis, the hazard ratios observed above did not change substantially. Increasing the time of drug use from one year minimal to 2 years, we still found that people who used CCBs had increased risk of total cancer (HR:1.07, 95%CI: 1.00–1.14), and people who used ARBs had increased risk of thyroid cancer (HR:1.58, 95%CI: 1.14–2.21). Cohort members who took two classes of antihypertensives also had an increased risk for total cancer (HR:1.13, 95%CI: 1.00–1.28). We also observed no changes in our results when including SBP as a continuous covariate in multivariate analysis.

## Discussion

In this population-based cohort study, we found certain types of antihypertensive drugs were associated with cancer risk although the associations were inconsistent in 4 specific cancer sites. While ACEIs, BBs and diuretics were found to have no association with the risk of cancer, ARBs and CCBs were associated with an approximately 10% increase in the risk of total cancer, with no modification by SBP. ARBs were also associated with increased risks of lung and thyroid cancer. A dose–response relationship was suggested for the number of antihypertensive drug classes used in association with total cancer risk, but the relationship was not shown for the 4 specific cancer sites.

Our findings on ARBs were consistent with the results of a meta-analysis of randomized controlled trials which also suggested modestly increased risks for total cancer [risk ratio (RR): 1.08, 95%CI: 1.01–1.15; *P* = 0.016] as well as for lung cancer (RR: 1.25, 95%CI: 1.05–1.49; *P* = 0.01) [6]. Although several other investigations did not find evidence to support these associations, those studies were different from ours in terms of their controls. They compared ARBs use to other antihypertensive drugs, such as ACEIs, which may not be an appropriate control group to compare with [9, 14]. It was also possible that different confounding variables could be responsible for this discrepancy. To our knowledge, this study was the first to report a possible association between thyroid cancer risk and ARBs use. A previous study found no association between ARBs use and risk of all endocrine cancers (RR: 0.83, 95%CI: 0.46–1.50, *P* = 0.54) [15]. Antihypertensive drugs were previously found to affect thyroid function, such as impacting thyroid hormone metabolism [16, 17].

There have been reports that BBs use was associated with increased risks of several types of cancer, including breast cancer [18]. Conversely, other researchers found use of BBs associated with reduced cancer risk or no associations with cancer [19], which was similar to our finding. CCBs were subjected to the same dilemma, as some studies found increased risk, but others showed reduced risk or no association [20, 21]. Interestingly, in our study we observed similar cancer risk for BBs and CCBs use, but the risk association with CCBs was statistically significant while the other was not (*P* = 0.055). We also found that CCBs were associated with the risk of total cancer, but no risk association was observed for any of the 4 common cancer sites.

The biological mechanisms linking antihypertensive drugs use to cancer risk are still unclear. ARBs, lowering blood pressure by blocking angiotensin-type 1 receptor, enable angiotensin-type 2 receptor for stimulation, which was reported to mediate tumor angiogenesis in vivo, particularly in lung tissue [22]. Evidence shows that activation of angiotensin-type 2 receptor enhances the activities of vascular endothelial growth factor and stimulates blood vessel formation, which facilitates cell proliferation [23]. BBs which inhibit the activity of beta adrenergic receptors may also play a role in tumor growth as the regimens can influence the downstream signaling pathway of cell cyclic adenosine monophosphate-protein kinase A which further affects tumor progression and metastasis. Some experiments indicated that CCBs could promote tumor growth by inhibiting apoptosis or interfering with cell differentiation through the calcium-related signals when elevated cytosolic calcium activates caspase or induces the endonuclease activity [24]. In addition to cytosolic calcium, lysosomal Ca2 + signaling can regulate the autophagy pathway to control cellular homeostasis [25]. Independent from the calcium channel effect, nifedipine affects the process of apoptosis through miRNA-524-5p-BRI3-extracellular signal-regulated kinase pathway [26]. Unfortunately, none of the above mechanisms have been verified in human, and therefore more studies are needed to further elucidate the possible mechanisms underlying these relationships.

In our study, participants with polytherapy were associated with a 22% elevated risk of cancer when comparing to those who never used major antihypertensive drugs while those with monotherapy seemed to show no cancer risk. Although previous studies explored the benefit of combined drug therapy in preventing cardiovascular disease and noticed the cancer risk of whether used specific antihypertensive drug or not, few researchers considered the cumulative cancer risks of using multi-classes of antihypertensive drugs [3]. Existing research was unable to provide reasonable mechanisms to explain the increased risk of using multiple drug classes. It remains uncertain whether the adverse events associated with increased number of blood pressure lowering medications are due to the accumulation of blood pressure reduction or the interaction of off-target effects. The highest hazards ratio for thyroid cancer in participants taking more than three classes of antihypertensives led to the concern over the drug’s effect on endocrine organs. Thyroid hormone levels varied under the treatment of several antihypertensives, with the renin–angiotensin–aldosterone system to enhance the capacity of thyroxine-binding, beta1-adrenergic blockers to mediate the effects of epinephrine and norepinephrine on pituitary and thyroid, and CCBs to inhibit intracellular availability of Ca^2+^ in thyroid cell, which might explain the strong association [27, 28].

Our study was a community-based study using a primary care database, which was based on a large community project aimed at hypertension management and control. Our study population can well represent the general hypertension patient population. We used hypertensive patients as the target population with adjustment for SBP level to reduce the possible surveillance bias and indication bias. However, there were several limitations that we should pay attention. First, dosage information on antihypertensive drugs taken by the participants is unavailable, and therefore we are unable to analyze the dose–response relationship to enhance the validity of our study findings. Further research on a dose–response relationship is warranted. Second, we only considered five common antihypertensive drugs in this study and did not include other drugs such as alpha-blockers. Nonetheless, the five antihypertensive drugs are the first-line medications for hypertension patients, covering most of the population who take medications for hypertension. Third, there may be other residual effects from unmeasured variables and residual confounding, although the statistical model was adjusted for several major covariates and confounding variables. Finally, the data of other commonly used drugs was unavailable and we were unable to assess the effect of other drugs such as metformin and statin. However, the cancer risk of metformin or statin were still controversial.

## Conclusion

In this population-based cohort study, the use of CCBs was associated with an increased risk of total cancer, while the use of ARBs was associated with an increased risk of total cancer, lung cancer, and thyroid cancer. Increasing usage of multiple antihypertensive classes was also associated with an increased cancer risk. Future studies involving larger populations and elucidating potential mechanisms will help to understand the adverse effects of antihypertensive drugs and develop better approaches to controlling hypertension while eliminating the risk of unintended consequences.

## Supplementary Information

**Additional file 1:** **Supplement Figure 1.** The venn diagram of the antihypertensive drug use.

**Additional file 2:** **Supplement Figure 2. **Hazards ratio for total and specific cancer associated with ever use of antihypertensive drugs stratified by SBP. HR=hazard ratio. *Adjusted for age, sex, body mass index, cigarette smoking, alcohol drinking, physical activity, diabetes and coronary artery heart disease. Chronic obstructive pulmonary disease included in lung cancer.

## Data Availability

Data available on reasonable request from the corresponding author.

## Abbreviations

ACEIs: Angiotensin-converting enzyme inhibitors
ARBs: Angiotensin receptor blockers
BBs: Beta blockers
CCBs: Calcium channel blockers
ICD10: The 10th International Classification of Diseases
SBP: Systolic blood pressure
BMI: Body mass index
CHD: Coronary artery heart disease
COPD: Chronic obstructive pulmonary disease
