# Supplementary figures and images for: Association between use of antihypertensive drugs and the risk of cancer: a population-based cohort study in Shanghai
Source: BMC Cancer. 2023 May 11;23:425. doi: 10.1186/s12885-023-10849-8 (PMC10173582; doi:10.1186/s12885-023-10849-8)

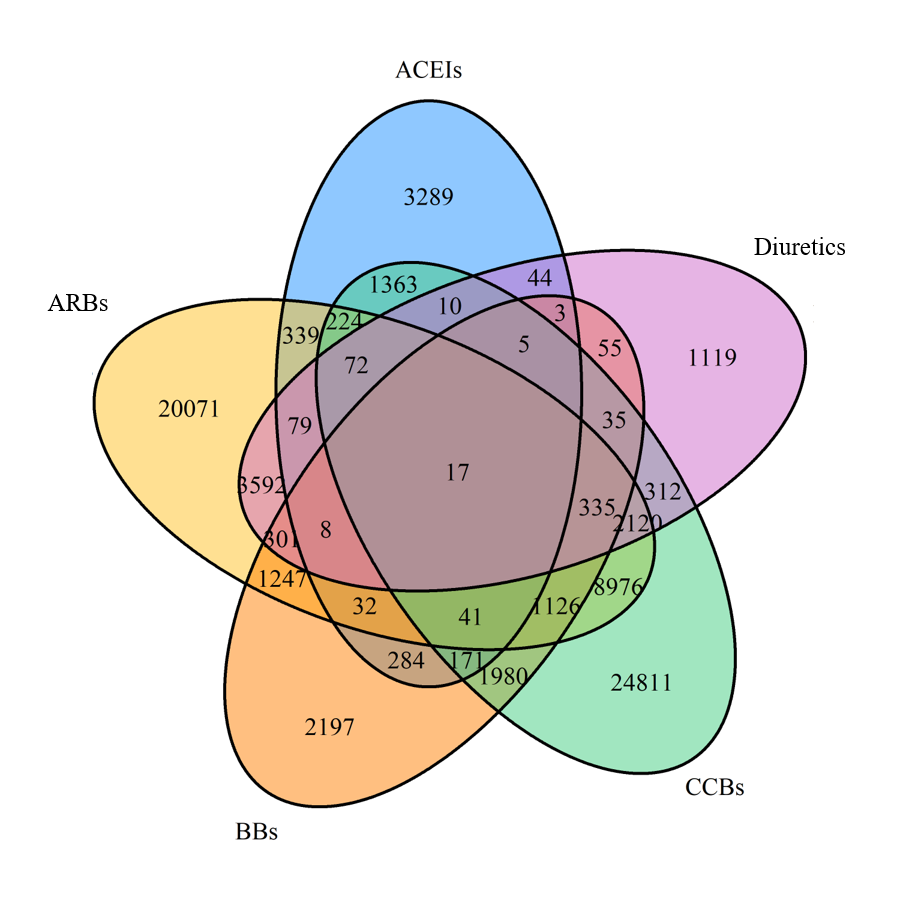

Supplement: Supplementary file 1 — Additional file 1: Supplement Figure 1. The venn diagram of the antihypertensive drug use. [file 12885_2023_10849_MOESM1_ESM.png]

SBP<130 mmHg

SBP≥130 mmHg

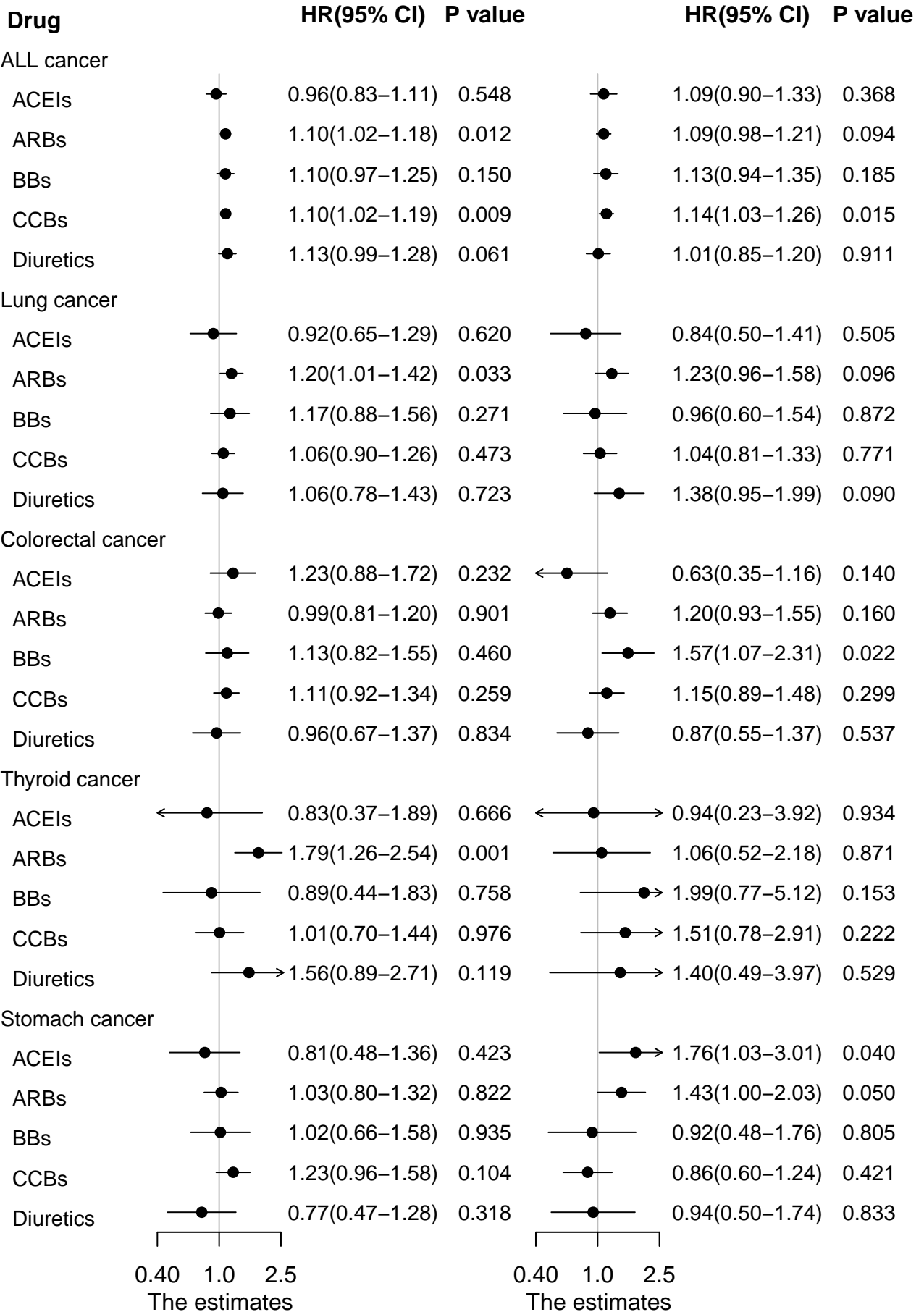

Supplement: Supplementary file 2 — Additional file 2: Supplement Figure 2. Hazards ratio for total and specific cancer associated with ever use of antihypertensive drugs stratified by SBP. HR=hazard ratio. *Adjusted for age, sex, body mass index, cigarette smoking, alcohol drinking, physical activity, diabetes and coronary artery heart disease. Chronic obstructive pulmonary disease included in lung cancer. [file 12885_2023_10849_MOESM2_ESM.pdf]
